# Selenomethionine Counteracts T-2 Toxin-Induced Liver Injury by Mitigating Oxidative Stress Damage Through the Enhancement of Antioxidant Enzymes

**DOI:** 10.3390/antiox14070866

**Published:** 2025-07-15

**Authors:** Yan Wang, Mingxia Zhou, Suisui Gao, Pishun Li, Xiaofeng Zheng, Di Tu, Lingchen Yang

**Affiliations:** 1 College of Veterinary Medicine, Hunan Agricultural University, Changsha 410128, China; wangy@hunau.edu.cn (Y.W.);; 2 Hunan Engineering Technology Research Center of Veterinary Drugs, Hunan Agricultural University, Changsha 410128, China

**Keywords:** selenomethionine, T-2 toxin, oxidative stress, mitochondrial biogenesis, antioxidant enzymes

## Abstract

T-2 toxin, a highly toxic feed contaminant, poses a significant health risk to both humans and animals, particularly targeting the liver. This study aimed to investigate the protective effects and underlying mechanisms of selenomethionine (SeMet) against T-2-induced liver injury in mice. We pretreated mice with SeMet before exposing them to an acute liver injury model induced by T-2. By assessing indicators related to liver injury, oxidative stress, inflammatory response, and mitochondrial disorder, we found that SeMet mitigated T-2-induced liver damage. Specifically, SeMet upregulated the gene expression and activity of antioxidant enzymes like glutathione peroxidase 1 (GPX1), which consequently reduced reactive oxygen species (ROS), inflammatory cytokines levels, and normalized mitochondrial biogenesis. Conclusively, SeMet effectively alleviated T-2-induced mitochondrial overproduction, inflammatory responses, and oxidative stress damage in hepatocyte primarily by enhancing GPX1 and other antioxidant enzymes, thereby exerting a protective effect on the liver. This study provides theoretical and experimental support for further research and application of SeMet in the livestock industry.

## 1. Introduction

Mycotoxins, toxic secondary metabolites ubiquitous in nature, represent a serious threat to animal health and food safety due to their contamination of grains and animal feeds [1]. T-2 toxin (T-2), a particularly potent type-A trichothecene, is predominantly synthesized by *Fusarium tricinctum* and *Fusarium poae* [2]. T-2 exhibits extremely stable physicochemical properties and resists complete inactivation or removal during food and feed processing, thus posing a persistent hazard to both humans and animals [3,4]. In recent years, T-2 contamination has occurred frequently globally, with particularly severe contamination reported in Chinese feed ingredients [4]. Therefore, investigating the toxic effects of T-2 and identifying effective antagonists is of great significance for ensuring food safety and animal health.

Upon ingestion by humans and animals, T-2 toxin not only causes poisoning symptoms, including nausea, vomiting, abdominal pain, diarrhea, and tachycardia [5], but also inflicts severe damage to the blood, digestive, immune, nervous, and reproductive systems [4,5,6]. Although T-2 is rapidly metabolized and cleared from the body, its toxicity markedly increases during enterohepatic circulation [7]. The liver, serving as the primary site of metabolism in organisms, as well as a crucial organ for digestion and detoxification, can be damaged by T-2 through various mechanisms, including oxidative stress induction and activation of inflammatory pathways [1,3]. These toxic effects impair normal liver functions and contribute to severe health issues, including liver cirrhosis and liver cancer [8,9]. Therefore, identifying effective drugs or nutritional supplements to counteract T-2-induced liver injury has emerged as a critical research focus.

Selenium (Se) is an essential micronutrient vital for absorption, metabolism, growth, immunity, and clinical health in animals. Supplementing livestock and poultry feed with appropriate doses of Se is crucial for animal well-being [10,11]. Se is also a key component of various antioxidant enzymes [12], capable of scavenging reactive oxygen species (ROS) radicals in the body and alleviating liver damage caused by oxidative stress [13]. Appropriate Se supplementation enhances antioxidant capacity, improves liver fibrosis, modulates mitochondrial number and volume, and can partially prevent hepatitis, liver necrosis, and liver cancer [10,13,14,15]. In nature, selenium exists mainly in two forms: inorganic selenium and organic selenium. Inorganic selenium primarily consists of selenium compounds such as selenite and selenate, which have the disadvantage of being relatively toxic. Organic selenium primarily includes selenium-containing amino acids, selenoproteins, selenium yeast, etc. In comparison with inorganic selenium, organic selenium demonstrates higher bioactivity and utilization, alongside significantly greater safety [11,16,17].

Selenomethionine (SeMet), a selenium-containing amino acid, is intricately linked to the organism’s redox mechanisms [18]. Research on the effects of various selenium sources on fluoride-induced liver damage has shown that SeMet is the optimal selenium supplement for mitigating fluorosis in mice [16]. Studies have shown that SeMet protects animals from aflatoxin B1 (AFB1)-induced oxidative damage by activating the Nrf2/HO-1-signaling pathway [19]. Furthermore, SeMet can modulate inflammatory responses by inhibiting the nuclear factor-κB (NF-κB) pathway and downregulating hepatic inflammatory cytokines, thus mitigating liver inflammation [14]. However, the precise capacity of SeMet to alleviate T-2-induced liver damage, along with the underlying mechanism responsible for its protective effect, is still not fully elucidated.

This study aimed to investigate the protective effects of SeMet on T-2-induced liver injury in mice and to elucidate the underlying molecular mechanisms. Mice were pretreated with SeMet prior to T-2-induced acute liver injury. By analyzing changes in liver function indicators, histopathological alterations, ROS content, and the expression levels of oxidative stress and inflammation-related genes in the liver, we sought to elucidate the mechanisms involved. The findings of this study are anticipated to offer a theoretical foundation for further research and scientific application of SeMet in livestock and poultry breeding, as well as provide new insights and scientific methodologies for preventing and treating T-2-induced toxicity.

## 2. Materials and Methods

### 2.1. Chemicals and Reagents

We bought T-2 toxin (purity ≥ 98%) from Sigma-Aldrich Company, Ltd. (Dorset, UK); DL-Selenomethionine (purity > 98%) from Beijing Solarbio Science & Technology Co., Ltd. (Beijing, China); N-Acetyl-L-cysteine (NAC) from Shanghai Macklin Biochemical Co., Ltd. (Shanghai, China), For biochemical assays, the Alanine aminotransferase (ALT) assay kit, Aspartate aminotransferase (AST) assay kit, Alkaline phosphatase (ALP) assay kit, Total protein (TP) assay kit, and Albumin (ALB) assay kit were all purchased from Shenzhen Mindray Bio-Medical Electronics Co., Ltd., (Shenzhen, China). For oxidative stress analysis, the Malondialdehyde (MDA) assay kit, Glutathione (GSH) assay kit, Superoxide dismutase (SOD) assay kit, and Lactate dehydrogenase (LDH) assay kit were obtained from Nanjing Jiancheng Bioengineering Institute (Nanjing, China), while the ROS staining solution was purchased from Wuhan Servicebio Technology Co., Ltd. (Wuhan, China).

### 2.2. Animals and Experimental Design

All animal experiments were performed in accordance with Animal Research: Reporting of In Vivo Experiments (ARRIVE) guidelines and the National Research Council’s “Guide for the Care and Use of Laboratory Animals” to ensure the scientific and ethical nature of the experimental operations. Furthermore, the use of mice and the experimental protocol were formally approved by the Animal Use and Care Ethics Committee of Hunan Agricultural University (HAUCEC2024-169). The sample size was calculated with Lamorte’s Power Calculations. In this experiment, 60 healthy SPF-grade Kunming strain male mice (aged 5–6 weeks, purchased from Changsha Tianqin Biotechnology Co., Ltd., Changsha, China) were randomly divided into six groups (*n* = 10) after a 1-week pre-feeding period. The experimental groups included: the control group (NC), the T-2 challenge group (T-2), and groups combining SeMet with T-2 (LG + T-2, MG + T-2, HG + T-2), as well as a group combining NAC with T-2 (NAC + T-2).

NC Group: Mice were gavaged with PBS (0.3 mL/day) for 14 days. Twelve hours after the last gavage, the mice were intraperitoneally injected with a mixture of 0.1 mL of ethanol and PBS. T-2 Group: Mice were gavaged with PBS (0.3 mL/day) for 14 days. Twelve hours after the last gavage, the mice were intraperitoneally injected with T-2 at a dose of 4 mg/kg body weight (B.W.). LG + T-2, MG + T-2, HG + T-2 Groups: Mice were gavaged with SeMet at doses of 0.25 mg/kg B.W., 0.5 mg/kg B.W., and 0.75 mg/kg B.W., respectively, once daily for 14 days. Twelve hours after the last gavage, the mice were intraperitoneally injected with T-2 at a dose of 4 mg/kg B.W. NAC + T-2 Group: Mice were gavaged with 0.3 mL NAC at a dose of 100 mg/kg B.W. once daily for 14 days. Twelve hours after the last gavage, the mice were intraperitoneally injected with T-2 at a dose of 4 mg/kg B.W. Twelve hours after intraperitoneal injection, the mice were anesthetized by isoflurane inhalation through a mask and dissected for sampling.

During the experiment, all mice were housed in the Animal Feeding Center of Hunan Agricultural University, following standard feeding and management practices, with access to a complete diet, free water, and food. The feeding environment was maintained at a constant temperature (20–25 °C) and humidity (50–70%), with a 12 h light/dark cycle. The mice were weighed every morning at 8:00, and their cages were regularly disinfected and cleaned. Additionally, the mice’s mental status, activity, coat, feces, food intake, and body weight were observed and recorded.

### 2.3. Sample Collection and Preparation

The blood sample was collected by ocular bleeding and then centrifuged at 3000 rpm for 10 min at 4 °C to obtain blood serum. The serum was used to detect the levels of ALT, AST, ALP, TP, ALB, and GLB. The liver tissue was collected and rinsed with pre-cooled physiological saline. Excess water on the tissue surface was absorbed with gauze. The wet weight of the liver was recorded, and the liver organ coefficient was calculated using the following formula:

Liver coefficient = Wet weight of liver/Body weight.

After it was photographed and weighed, the liver tissue was cut into 5 × 5 × 3 mm blocks and fixed in 4% paraformaldehyde solution for subsequent histopathological and immunohistochemical analysis. The remaining liver tissue was cut and portioned, quickly frozen in liquid nitrogen, and then kept in a −80 °C freezer for following experiments.

### 2.4. Histopathological Analysis

The liver tissue fixed in 4% paraformaldehyde was processed for paraffin sectioning, dewaxing, and hydration. The sections were fixed, followed by hematoxylin staining, eosin staining, dehydration, and mounting. Finally, the sections were examined under a microscope, and images were captured and analyzed.

### 2.5. Serum Biochemical Analysis

The serum samples were analyzed on automatic biochemical analyzers using commercial kits to detect the concentration of ALT, AST, ALP, TP, and ALB in accordance with the instructions. The level of globulin (GLB) was calculated using the formula: GLB = TP − ALB. The ratio of A/G was calculated using the formula: A/G = ALB/GLB.

### 2.6. Hepatic Biochemical Analysis

Different oxidative stress-related indicators in liver tissue were analyzed with specific commercial reagent kits, and the results were normalized to the protein concentration. The protein concentration was detected using BCA (Bicinchoninic Acid) assay in accordance with the instructions provided in the commercial kit. The concentration of MDA and GSH, as well as the activity of LDH, CAT, and SOD, was measured and calculated following the instructions. The OD values were measured with a microplate reader.

### 2.7. ROS Content Analysis

ROS content analysis was conducted using a commercial kit according to the instructors. After the frozen sections were brought to room temperature and dried. A spontaneous fluorescence quencher was added for 5 min, followed by rinsing with running water for 10 min. Next, the sections were incubated with ROS staining solution (DCFH-DA) at 37 °C in the dark for 30 min. The nuclei were counterstained with 4′,6-diamidino-2-phenylindole (DAPI) after PBS wash. Then, the slides were mounted with an anti-fluorescent quenching. Images were visualized with a fluorescence microscope. Quantitative analysis of the acquired immunofluorescence images was performed by densitometry using ImageJ software 1.6.0 (National Institutes of Health, Bethesda, MD, USA).

### 2.8. Real-Time qPCR Analysis

To investigate the mRNA relative expression of inflammation and oxidative stress-related genes, specific primers were designed based on relevant gene sequences. Detailed information is listed in Table S1.

RNA extraction and real-time qPCR (RT-qPCR) were performed as previously described with modifications [20]. Briefly, 50 mg of liver tissue were homogenized in Trizol reagent (Takara Co., Otsu, Shiga, Japan) using a biological sample homogenizer to isolate total RNA. Next, Qualified RNA was reverse transcribed using a one-step method (Hifair II first Strand cDNA Synthesis SuperMix, Yeasen Biotechnology Co., Ltd., Shanghai, China) according to the manufacturer’s instructions. The cDNA synthesized by reverse transcription was used as the template for the real-time qPCR with ChamQ universal SYBR qPCR Master Mix (Vazyme Biotechnology Co., Ltd., Nanjing, China) on an ABI 7300 (Applied Biosystems, Foster City, CA, USA). The relative mRNA expression levels of the target genes were normalized with the reference gene (GAPDH) using the 2^−ΔΔCt^ method.

### 2.9. Immunohistochemistry Analysis

Immunohistochemistry (IHC) was performed to detect the expression of inflammatory response and mitochondrial stress-related proteins. The liver samples were processed through paraffin sectioning, antigen retrieval and washing, inhibition of endogenous enzyme activity, serum blocking, primary antibody incubation, secondary antibody binding, diaminobenzidine (DAB) staining, nuclear counterstaining, dehydration, mounting, and, finally, interpreted under a light microscope. ImageJ software 1.6.0 was used for quantitative analysis of the positive signals in the immunohistochemistry-stained sections.

### 2.10. Statistical Analysis

Statistical analysis was done with SPSS Statistics 26.0 software (IBM Inc., New York, NY, USA). One-way ANOVA was performed for all groups, and the LSD test was used for multiple comparisons. The normality of the distribution of the variables was assessed before statistical analysis. The experiments were repeated three times, and the results are expressed as the mean ± standard error. *p* < 0.05 was indicated as statistically significant. Figures were obtained using GraphPad Prism 9.5 (Graphpad Prism Inc., San Diego, CA, USA) and Adobe Illustrator 2020 (Adobe Systems, San Jose, CA, USA).

## 3. Results

### 3.1. Effects of SeMet on Food Intake, Body Weight, and Liver Coefficient of T-2 Toxin-Exposed Mice

**Figure 1:**
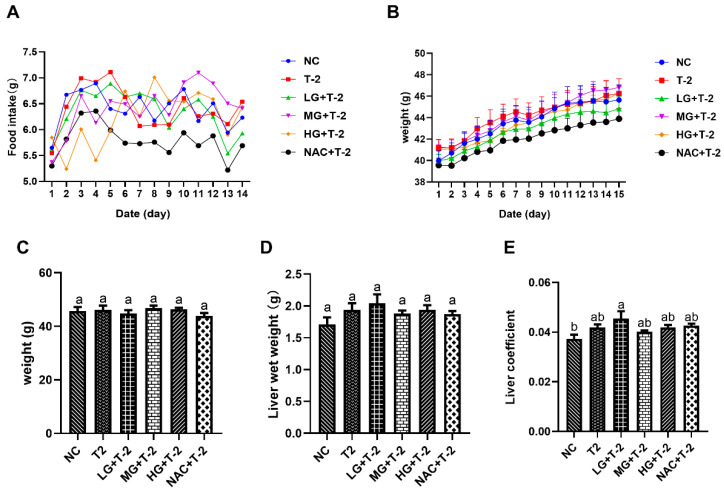
The effect of selenomethionine on body weight, food intake, and liver coefficient in T-2 toxin-exposed mice. (**A**) Trend of food intake and (**B**) weight gain over time among mice in different groups. (**C**) Body weight, (**D**) liver wet weight, and (**E**) liver coefficient of mice in all groups. NC, control group; T2, T-2 toxin group; LG + T-2, MG + T-2, HG + T-2, 0.25, 0.5, and 0.75 mg/kg SeMet combined with T-2 toxin group; NAC + T-2, N-acetylcysteine combined with T-2 group. Different letters indicated significant differences, *p* < 0.05.

Figure 1A,B illustrated the time-dependent changes in food intake and body weight throughout the experimental period. The results indicated that the average daily weight gain across all groups remained within 1 g, reflecting normal growth. Additionally, average daily food intake ranged from 5 to 7.5 g across all groups, which is within the normal range. These findings suggested that the SeMet gavage and T-2 exposure did not significantly impact food intake or body weight in present study. Figure 1C,D indicated no significant differences in body weight or liver wet weight among all groups (*p* > 0.05). However, the liver coefficient in the LG + T-2 group was significantly higher than that in NC group (*p* < 0.05), while no significant differences were observed in the liver coefficients of the other groups compared to the NC group (Figure 1E, *p* > 0.05).

### 3.2. Protective Effects of SeMet on Liver Morphology and Function in T-2 Toxin-Exposed Mice

**Figure 2:**
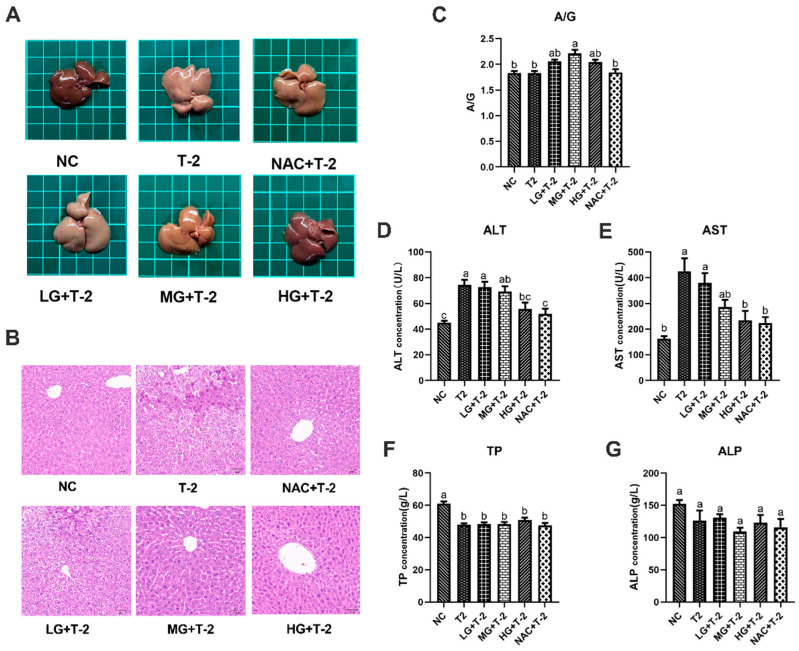
The effect of selenomethionine on the morphology and function of liver in T-2 toxin-exposed mice. (**A**) Morphological observation, (**B**) histopathological analysis of liver in mice. (**C**) The ratio of A/G, the concentration of (**D**) ALT, (**E**) AST, (**F**) TP, and (**G**) ALP in the serum of mice in all groups. Different letters indicated significant differences, *p* < 0.05.

As depicted in Figure 2A, the liver of NC group mice exhibited a reddish-deep color, whereas T-2 group mice displayed dull yellow livers with noticeable pinpoint protrusions on the surface. Compared to the T-2 group, the liver color in the LG + T-2, MG + T-2, and HG + T-2 groups gradually shifted back to a reddish hue, accompanied by a reduction in surface protrusions. Notably, the liver morphology in the HG + T-2 group most closely resembled that of the NC group. The liver of NAC + T-2 group was pinkish, with clear edges and normal structure. These findings suggested that both NAC and SeMet could mitigate T-2-induced morphological alterations in the liver, with high-dose SeMet demonstrating the most significant improvement.

Histopathological analysis revealed that the NC group exhibited well-organized hepatocyte cords and sinusoids with normal cell morphology. After T-2 toxin challenge, the normal liver structure was disrupted, with hepatocytes exhibiting granular degeneration, nuclear fragmentation, necrosis, and disintegration. In the LG + T-2 group, disordered hepatocyte cords, hepatocyte swelling, granular degeneration, and partial necrosis were observed. The MG + T-2 group showed restored liver cord structure but also dilated perisinusoidal spaces, hepatocyte atrophy, increased nucleocytoplasmic ratio, prominent nucleoli, frequent binucleated hepatocytes, and increased Kupffer cells in the perisinusoidal spaces compared to the NC group. The HG + T-2 group exhibited neatly arranged hepatocyte cords, mild congestion, dilation of perisinusoidal spaces, and an overall normal liver structure without pathological abnormalities. The NAC + T-2 group, as the antioxidant control, showed with dilated and congested perisinusoidal spaces, frequent binucleated hepatocytes, and scattered necrotic hepatocytes and inflammatory cells. These results suggested that T-2 disrupted normal liver architecture, leading to inflammatory cell infiltration and extensive cell necrosis. However, medium and high doses of SeMet could effectively reduce the elevation of inflammatory cells and necrotic cells, thus protecting the normal liver structure. SeMet exhibited a dose-dependent protective effect against T-2-induced hepatocellular damage (Figure 2B).

Serum biochemical markers related to liver function were subsequently assessed. Results revealed that T-2 challenge significantly increased the concentrations of ALT and AST, decreased the concentration of TP (*p* < 0.05), but showed no significant effects on the ratio of A/G and ALP (*p* > 0.05). However, SeMet supplementation alleviated T-2-induced liver dysfunction to some extent (Figure 2C–G). Specifically, compared to the NC group and the T-2 group, only the ratio of A/G in the MG + T-2 group showed a significant increase (*p* < 0.05), with no significant differences in other groups (*p* > 0.05). Compared to the NC group, the ALT concentrations in the T-2, LG + T-2, and MG + T-2 groups were significantly increased (*p* < 0.05), while there were no significant differences in the HG + T-2 and NAC + T-2 groups (*p* > 0.05). Compared to the T-2 group, there were no significant differences in the LG + T-2 and MG + T-2 groups (*p* > 0.05), but the ALT concentrations in the HG + T-2 and NAC + T-2 groups were significantly decreased (*p* < 0.05). Compared to the NC group, the AST concentrations in the T-2 and LG + T-2 groups were significantly increased (*p* < 0.05), with no significant differences in the MG + T-2, HG + T-2, and NAC + T-2 groups (*p* > 0.05). Compared to the T-2 group, the AST concentrations in the HG + T-2 and NAC + T-2 groups were significantly decreased (*p* < 0.05), with no significant differences in the LG + T-2 and MG + T-2 groups (*p* > 0.05). Compared to both the NC and T-2 groups, there were no significant differences in ALP concentrations among the groups (*p* > 0.05). Compared to the NC group, the TP content in the liver tissue of all groups was significantly decreased (*p* < 0.05). Compared to the T-2 group, there were no significant differences in TP content among the other groups (*p* > 0.05).

### 3.3. Effects of SeMet on T-2 Toxin-Induced Oxidative Stress in Mouse Livers

**Figure 3:**
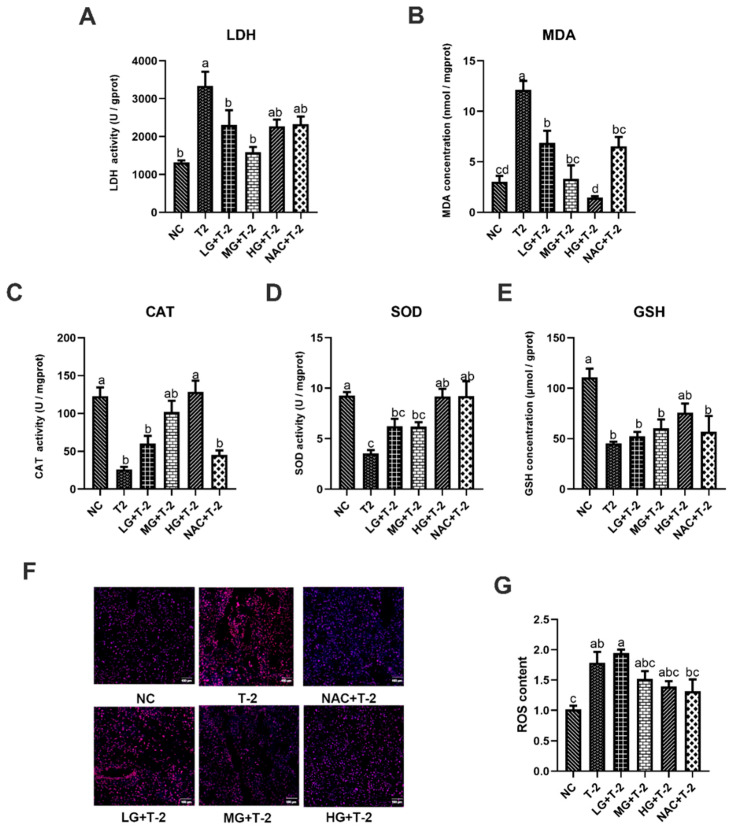
The effect of selenomethionine on oxidative stress induced by T-2 toxin in livers of mice. (**A**) LDH activity, (**B**) MDA concentration, (**C**) CAT activity, (**D**) SOD activity, (**E**) GSH concentration, (**F**) ROS fluorescence staining, and (**G**) ROS content in mice liver challenged by T-2 with SeMet intervention. Scale bar = 100 μm. Values with difference superscripts are significant differences (*p* < 0.05).

In comparison with the NC group, T-2 challenge significantly elevated LDH activity and MDA concentration while reducing CAT and SOD activities, as well as GSH concentration (*p* < 0.05). The SetMet supplement markedly enhanced the antioxidant capacity and alleviated T-2 toxin-induced oxidative stress damage in the liver in a dose-dependent manner. Specifically, compared with the T-2 group, 0.25 and 0.5 mg/kg of SeMet significantly decreased LDH activity and MDA concentration (*p* < 0.05) but only showed a non-significant increase in CAT, SOD activity, and GSH concentration. Further, 0.75 mg/kg of SeMet significantly decreased MDA concentration and increased CAT and SOD activity (*p* < 0.05), while the reduction in LDH activity and elevation in GSH concentration was not statistically significant. NAC treatment significantly reduced MDA concentration and elevated SOD activity (*p* < 0.05), but the decrease in LDH activity and increase in CAT activity and GSH concentration are not significant (Figure 3A–E).

Figure 3F,G illustrate the results of ROS fluorescence staining and ROS content in mouse liver tissue induced by T-2 with SeMet intervention. Results showed that compared to the NC group, ROS content in the liver tissue of the T-2 and LG + T-2 groups significantly increased (*p* < 0.05), while there were no significant differences in the MG + T-2, HG + T-2, and NAC + T-2 groups. When compared to the T-2 group, although there were no significant differences in ROS content across groups (*p* > 0.05), a decreasing trend was observed in the MG + T-2, HG + T-2, and NAC + T-2 groups. This indicated that T-2 could lead to a significant accumulation of ROS in the liver, increasing its content, while both SeMet and NAC could reduce ROS levels in the liver.

### 3.4. Modulation of Inflammation and Oxidative Stress-Related Gene Expression by SeMet in T-2 Toxin-Exposed Mice Livers

**Figure 4:**
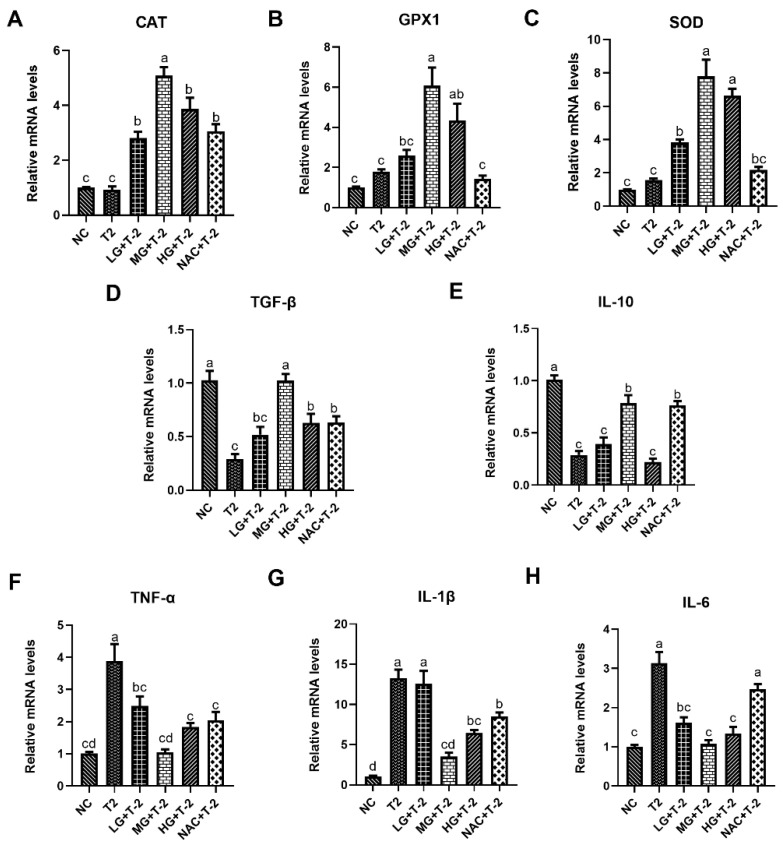
The effect of selenomethionine on T-2 toxin-induced gene expression changes related to inflammation and oxidative stress in livers of mice. The relative mRNA levels of antioxidant enzymes (**A**) *CAT*, (**B**) *GPX1*, (**C**) *SOD*, and inflammatory response-related genes (**D**) *TGF-β*, (**E**) *IL-10*, (**F**) *TNF-α*, (**G**) *IL-1β*, and (**H**) *IL-6* in mice liver challenged by T-2 with SeMet intervention. Different letters indicated significant differences, *p* < 0.05.

Figure 4A–C illustrated the relative mRNA expression levels of antioxidant enzymes in mouse liver tissue induced by T-2 with SeMet intervention. Compared with the NC group, the T-2 group exhibited a slight upward trend in the relative mRNA levels of *CAT*, glutathione peroxidase 1 (*GPX1*), and *SOD*. Meanwhile, supplementation with SeMet could significantly upregulate the gene expression of antioxidant enzymes in a dose-dependent manner. When compared with the T-2 group, the relative mRNA levels of *CAT* were significantly upregulated in the LG + T-2, MG + T-2, HG + T-2, and NAC + T-2 groups (*p* < 0.05). The relative mRNA levels of *GPX1* were significantly upregulated in the MG + T-2 and HG + T-2 (*p* < 0.05) groups. The relative mRNA levels of *SOD* were significantly upregulated in the LG + T-2, MG + T-2, and HG + T-2 groups (*p* < 0.05).

Figure 4D–H depictd the relative mRNA expression levels of genes related to inflammatory response in mouse livers after T-2 challenge and SeMet intervention. Compared to the NC group, T-2 exposure significantly downregulated the relative mRNA levels of *TGF-β* and *IL-10* and upregulated the relative mRNA levels of *TNF-α*, *IL-1β,* and *IL-6* (*p* < 0.05). When compared with the T-2 group, the LG + T-2 group showed a significant decrease in the gene expression of *TNF-α* and *IL-6* (*p* < 0.05); the MG + T-2 group displayed a significant increase in the expression levels of *TGF-β* and *IL-10*, as well as a significant decrease in the expression levels of *TNF-α*, *IL-1β,* and *IL-6* (*p* < 0.05); the HG + T-2 group exhibited a significant increase in the gene expression of *TGF-β* and a significant decrease in the gene expression of *TNF-α*, *IL-1β,* and *IL-6* (*p* < 0.05). Moreover, NAC treatment showed similar anti-inflammatory response effects, accompanied by a significant upregulation in the relative mRNA levels of *TGF-β* and *IL-10*, as well as a downregulation in the relative mRNA levels of *TNF-α* and *IL-1β* (*p* < 0.05).

### 3.5. Impact of SeMet on IL-1β and TNF-α Expression in the Livers of T-2 Toxin-Exposed Mice

**Figure 5:**
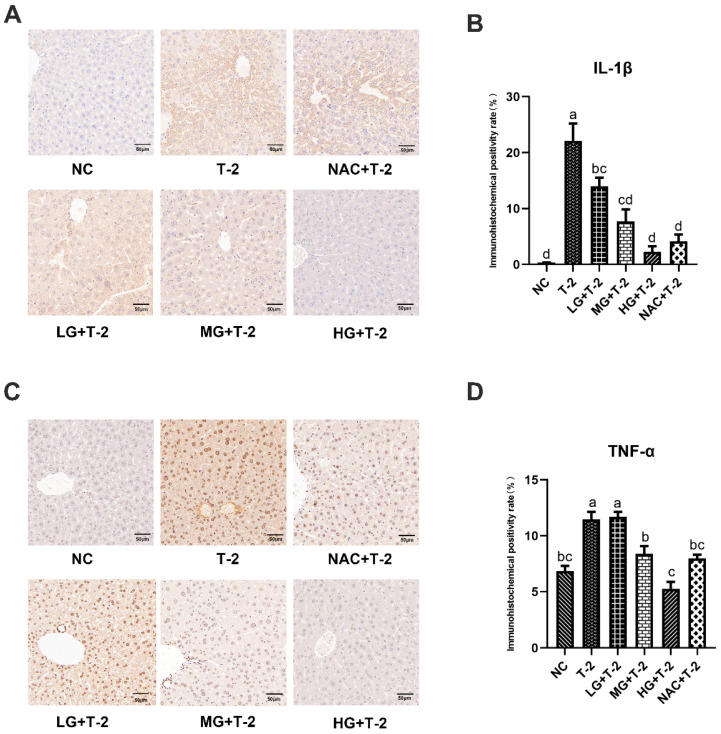
The effect of selenomethionine on the immunohistochemical morphology and positivity rate of inflammatory cytokine proteins in the liver of T-2 toxin-induced mice. (**A**) The immunohistochemical section of IL-1β and (**B**) quantitative results of positive rate, (**C**) the immunohistochemical images of TNF-α, and (**D**) quantitative results of positive rate in the liver of mice challenged by T-2 with SeMet intervention. The scale bar = 50 μm. Different letters indicated significant differences, *p* < 0.05.

Figure 5A,B displayed that, compared with the NC group, the IL-1β protein was highly expressed in hepatocytes surrounding the central venous endothelium in the T-2 group. Compared with the T-2 group, as the SeMet dose increased, the number of positive hepatocytes with IL-1β protein expression around the central venous endothelium gradually decreased, and the HG + T-2 group was closest to the NC group. The number of positive hepatocytes in the NAC + T-2 group was lower than that in the T-2 group but still higher than that in the HG + T-2 group. The statistical analysis results further revealed that the IL-1β positive expression rate in the T-2 group was significantly higher (*p* < 0.05) than that of the NC group. Compared with the T-2 group, the IL-1β positive expression rate in all groups significantly decreased (*p* < 0.05), with the HG + T-2 group having the lowest IL-1β positive expression rate.

Figure 5C,D demonstrated that, compared with the NC group, TNF-α protein was highly expressed in the hepatocyte membranes and extracellular stroma in the T-2 group. Compared with the T-2 group, the number of hepatocytes with positive TNF-α protein expression decreased in all groups, with the HG + T-2 group being closest to the NC group. Statistical analysis results showed that the TNF-α positive expression rate significantly increased in the T-2 group (*p* < 0.05) than that of the NC group. Compared with the T-2 group, the TNF-α positive expression rate significantly decreased in the MG + T-2 group, the HG + T-2 group, and the NAC + T-2 group (*p* < 0.05), with the HG + T-2 group having the lowest TNF-α positive expression rate. However, there was no significant difference in the LG + T-2 group (*p* > 0.05).

### 3.6. Effect of SeMet on the Immunohistochemical Expression of Mitochondrial Homeostasis-Related Proteins in the Livers of T-2 Toxin-Induced Mice

**Figure 6:**
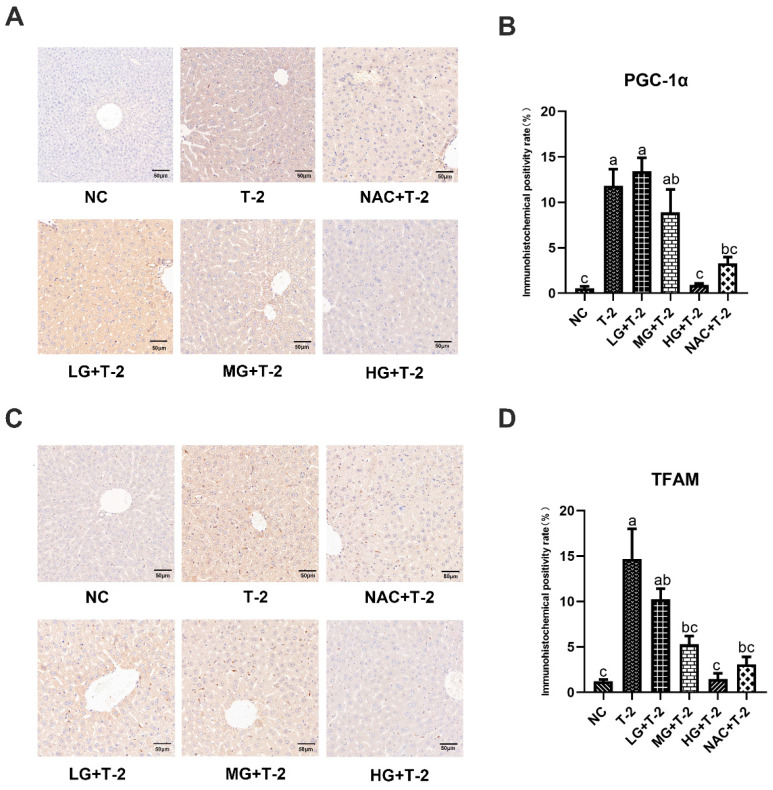
The effect of selenomethionine on the immunohistochemical morphology and positivity rate of mitochondrial homeostasis-related proteins in the liver of T-2 toxin-induced mice. (**A**) The immunohistochemical section of PGC-1α and (**B**) quantitative results of positive rate, (**C**) the immunohistochemical images of TFAM, and (**D**) quantitative results of positive rate in the liver of mice challenged by T-2 with SeMet intervention. The scale bar = 50 μm. Different letters indicated significant differences, *p* < 0.05.

Compared with the NC group, the peroxisome proliferator-activated receptor γ coactivator-1α (PGC-1α) protein was highly expressed in Kupffer cells within the sinusoidal spaces in the T-2 group. Compared with the T-2 group, the number of positive cells decreased in the MG + T-2 group, the HG + T-2 group, and the NAC + T-2 group, with the HG + T-2 group being closest to the NC group (Figure 6A). The statistical analysis results further confirmed that the PGC-1α positive expression rate significantly increased in the T-2 group (*p* < 0.05) than the NC group. Compared with the T-2 group, the PGC-1α positive expression rate significantly decreased in the HG + T-2 group and NAC + T-2 group (*p* < 0.05), while there were no significant differences in the LG + T-2 group and MG + T-2 group (Figure 6B, *p* > 0.05).

Compared with the NC group, mitochondrial transcription factor A (TFAM) protein was highly expressed in Kupffer cells within the sinusoidal spaces in the T-2 group. Compared with the T-2 group, the number of Kupffer cells with positive TFAM protein expression decreased in all groups, with the HG + T-2 group having the smallest number of positive Kupffer cells (Figure 6C). Figure 6D shows that the TFAM positive expression rate significantly increased in the T-2 group (*p* < 0.05) compared to the NC group. Compared with the T-2 group, the TFAM positive expression rate significantly decreased in the MG + T-2 group, the HG + T-2 group, and the NAC + T-2 group (*p* < 0.05), but there was no significant difference in the LG + T-2 group (*p* > 0.05).

## 4. Discussion

T-2 toxin, a potent type A trichothecene, is one of the most hazardous mycotoxins, commonly contaminating animal feed and posing significant health risks to both animals and humans [2,21,22]. Due to its widespread occurrence, T-2 toxin has attracted global attention for preventive measures in the grain and livestock sectors. As the primary site of metabolism in the body, the liver is also a primary target organ of T-2 [3]. Oxidative stress and inflammatory response are the primary mechanisms underlying T-2-induced liver toxicity [15,23]. Therefore, selecting appropriate antioxidants can effectively prevent the toxic effects of T-2 on the liver. In this study, SeMet was chosen as the selenium source to explore its protective effects against T-2-induced liver injury in mice and the underlying mechanism. Our findings revealed that SeMet supplementation effectively counteracted the inflammatory response, oxidative stress damage, and normalized mitochondrial overproduction in the liver exposed to T-2. This protective mechanism is related to SeMet’s enhancement of antioxidant enzymes.

Se is an essential trace element in the body with various biological functions, including anti-inflammatory and antioxidant properties [10]. Both Se deficiency and Se poisoning are harmful, with weight loss indicating toxicity in rodents [10,24]. Throughout the experimental period, the mice in all groups exhibited steady body weight gain, indicating no Se poisoning, and the selected doses of SeMet had no impact on food intake or mental status. We induced an acute liver injury model in mice with T-2 intraperitoneal injection and found no effect on body weight. The liver coefficient, an indicator for evaluating liver injury and function, reflects changes in liver morphology, which was calculated by comparing the body weights and liver wet weights [25]. Our results showed a non-significant upward trend in liver wet weight and liver coefficient across groups, possibly due to the short duration of T-2 exposure.

Although T-2 exposure had no significant effect on the liver coefficient, pathological examination revealed liver alterations in the T-2 group. The livers in the T-2 group exhibited clay-colored discoloration, loss of elasticity, and surface protrusions, along with extensive inflammatory infiltration and hepatocellular necrosis. These findings indicated that T-2 disrupted liver structure and induced inflammation and necrosis, thereby altering liver morphology and causing injury, which is consistent with earlier studies on T-2-induced hepatotoxicity [3]. SeMet supplementation, particularly at higher doses, ameliorated these morphological and structural abnormalities, as demonstrated by the recovery of hepatic cord structure and reduction of inflammatory cell infiltration. This aligns with previous findings that Se supplementation counteracts mycotoxin-induced liver injury [15].

ALT, AST, ALP, TP, and A/G ratio are crucial for liver function assessment [26]. Studies have shown that T-2 toxin exposure elevated AST, ALP, and ALT while lowering TP in broilers and broiler hepatocytes [9]. Similar results were obtained in this study, with the T-2 group exhibiting significantly elevated serum ALT and AST activities and decreased TP levels compared to the NC group, indicating T-2-induced hepatocyte damage and liver dysfunction. However, there was no significant difference in ALP levels between the T-2 and NC groups, potentially attributed to the duration of T-2 exposure; ALP may not be the most direct or sensitive marker of liver injury and may remain normal in early liver damage stages. ALP is typically used as a marker of cholestasis, with its levels closely related to bile duct injury. In contrast, AST and ALT are more sensitive indicators of hepatocellular damage, reflecting the disruption of hepatocyte membrane integrity and subsequent enzyme release into the bloodstream [27]. This finding suggested that the T-2 toxin primarily induced hepatocellular damage rather than cholestasis in our experiment. Therefore, despite the lack of significant changes in ALP levels, the significant elevation of AST and ALT clearly demonstrates the toxic effects of T-2 toxin on the liver. Although SeMet supplementation showed no significant difference in ALT and AST activities, a decreasing trend was noted, and the A/G ratio significantly increased in the MG + T-2 group, suggesting that SeMet can protect the liver from T-2 toxicity and restore normal liver function to some extent.

Oxidative stress is one of the primary mechanisms of T-2 toxicity [3,6,7]. External stimuli disrupt the body’s oxidative–antioxidant balance, causing ROS accumulation, which triggers lipid peroxidation and cellular damage. Increased MDA levels, the end product of lipid peroxidation, directly reflect the severity of cellular damage [28,29]. The study demonstrated that T-2 increased ROS levels, inducing oxidative stress in differentiated cells [30]. Additionally, multiple studies have shown that T-2 significantly elevates LDH activity in cells and tissues, causing damage [20,22,31]. This study consistently found that T-2 significantly elevated ROS and MDA levels, as well as LDH activity, in liver tissue. These findings suggested that T-2 impacts hepatocyte free radical metabolism, leading to excessive accumulation of ROS, which induces oxidative stress and cellular damage. In contrast, SeMet significantly decreased ROS and MDA levels, along with LDH activity, suggesting its ability to counteract T-2-induced ROS accumulation and alleviate oxidative stress damage, aligning with previous observations [15].

Inflammation is closely linked to oxidative stress [32] and is another major pathway of mycotoxin hepatotoxicity [15,23]. The study has shown that T-2 induced oxidative damage and promoted the expression levels of inflammation-related genes by activating JNK in IPEC-J2 cells [31]. Similar results were obtained in this study, with T-2 significantly upregulating pro-inflammatory cytokines (TNF-α, IL-1β, and IL-6) and downregulating anti-inflammatory markers (TGF-β, IL-10), promoting an inflammatory response in the liver. In a previous report, SeMet demonstrated a notable ability to counteract fluoride-induced inflammatory reactions in mice liver by downregulating pro-inflammatory factors and inhibiting the NF-κB-signaling pathway [16]. Consistent with this finding, our study revealed that SeMet supplementation attenuated T-2-induced inflammatory response by reducing TNF-α, IL-1β, and IL-6 expression while increasing TGF-β and IL-10 expression, particularly at higher doses. These results suggested that SeMet could effectively alleviate liver inflammatory damage caused by T-2 and restore normal liver structure and function.

Cellular oxidative stress is tightly linked to mitochondrial disorder [33]. Mitochondria, the cellular “energy factories”, generate ATP via oxidative phosphorylation and are primary sites of reactive oxygen species (ROS) production [34]. ROS levels increase in response to endogenous or exogenous stimuli, potentially causing mitochondrial structural damage and leading to cellular malfunction and cell death [33,35]. Peroxisome proliferator-activated receptor gamma coactivator 1α (PGC-1α) and mitochondrial transcription factor A (TFAM) are crucial regulators of energy metabolism, cellular stress, and mitochondrial function. PGC-1α serves as a pivotal transcription factor that modulates mitochondrial biogenesis and respiratory function [36]. As master coordinators of mitochondrial biogenesis, PGC-1α activated TFAM to drive replication of mitochondrial DNA and assembly of respiratory chain complexes, enhancing mitochondrial biogenesis [37]. Immunohistochemical analysis revealed a significant increase in the number of hepatocytes with positive PGC-1α and TFAM expression in the T-2 group, suggesting that T-2 can induce mitochondrial disorder and compensatory mitochondrial biogenesis in hepatocytes, consequently causing liver injury. The compensatory upregulation of PGC-1α and TFAM reflects a recognized stress response to mitochondrial dysfunction, triggered by stress-induced damage [38,39]. Similarly, endotoxin administration induced compensatory mitochondrial biogenesis, marked by increased PGC-1α expression, alongside mitochondrial dysfunction characterized by decreased O_2_ consumption and ATP production rate, ultimately leading to cardiac damage [40]. These findings are consistent with previous research that T-2 toxin upregulates PGC-1α and TFAM expression, inducing mitochondria overproduction and oxidative stress [38]. Critically, SeMet supplementation (especially at higher doses) effectively reduced PGC-1α/TFAM expression, normalized mitochondrial overproduction. This demonstrated SeMet’s modulatory action on mitochondrial biogenesis, thereby shielding liver cells from the deleterious effects of T-2. This protective effect of SeMet on mitochondrial health aligns with previous findings that nano-Se hindered the PGC1α pathway in chicken, alleviating cadmium-induced liver fibrosis [41]. However, the current study did not include direct mitochondrial functional assessments, which should be addressed in future investigations to further elucidate the functional consequences of SeMet intervention on mitochondria.

Antioxidant enzymes, such as SOD, CAT, and GPX, play essential roles in defending against oxidative stress by neutralizing ROS [42]. GSH, as a thiol-disulfide redox buffer, has its decreased levels linked to mitochondrial dysfunction and oxidative stress [5]. Multiple studies have shown that T-2 diminished the antioxidant capacity of the body by suppressing the activity of antioxidant enzymes SOD and CAT [6,9,30]. We also observed that T-2 significantly reduced GSH concentration, as well as SOD and CAT activity, compromising hepatic redox defense. Se primarily fulfills its biological role by integrating into selenoproteins as selenocysteine [10,43]. Se participates in the construction of several antioxidant enzymes, such as glutathione peroxidase (GSH-Px), a family of six selenoenzymes (GPX 1-6) [44]. Among these, GPX1 stands out as a vital antioxidant enzyme, responsible for scavenging peroxides and effectively mitigating oxidative stress-induced cellular damage within mitochondrial and cytosolic compartments [20]. Furthermore, research indicated that SeMet boosted antioxidant enzyme activity in the chicken brain by activating the NRF2/GPX4 pathway [43]. In this study, we also observed that SeMet significantly upregulated the expression levels of *GPX1*, *SOD,* and *CAT*. Additionally, it elevated GSH and SOD levels, as well as CAT activity, in liver tissue. The upregulation of GPX1, a mitochondria-localized antioxidant, highlighted SeMet’s mechanism of action. These findings suggested that SeMet enhanced the gene expression of GPX1 and boosted the activity of antioxidant enzymes in the liver, thereby reducing ROS production, alleviating mitochondrial disorder, and mitigating oxidative stress and inflammatory responses. Consequently, SeMet demonstrated a notable therapeutic effect against T-2-induced liver oxidative damage.

Conclusively, this study demonstrated that T-2 toxin induced liver damage in mice, accompanied by mitochondrial disorder, oxidative stress, and inflammatory responses. SeMet supplementation upregulated GPX-1 expression and enhanced antioxidant enzyme activity, effectively alleviating T-2-induced hepatotoxicity. The elevated expression levels and activity of these antioxidant enzymes collectively improved antioxidant capacity, decreased ROS production and accumulation, normalized mitochondrial overproduction, and mitigated oxidative stress and inflammatory response, thereby alleviating liver damage and dysfunction caused by T-2. Our findings provide theoretical and experimental support for the potential use of SeMet as a mycotoxin detoxifier in animal husbandry.

## Data Availability

The data of the research are available through the corresponding writer on reasonable demand.

## Supplementary Materials

The following supporting information can be downloaded at: https://www.mdpi.com/article/10.3390/antiox14070866/s1, Table S1: Primer sequences used for real-time quantitative PCR.

## Abbreviations

The following abbreviations are used in this manuscript: BMECs, bovine mammary epithelial cells; T-2, T-2 toxin; SeMet; selenomethionine; GPX1, glutathione peroxidase 1; ROS, reactive oxygen species; Se, selenium; AFB1, aflatoxin B1; NAC, N-Acetyl-L-cysteine; ALT, alanine aminotransferase; AST, aspartate aminotransferase; ALP, alkaline phosphatase; TP, total protein; ALB, albumin; MDA, malondialdehyde; GSH, glutathione; SOD, superoxide dismutase; LDH, lactate dehydrogenase; GLB, globulin; DAPI, 4′,6-diamidino-2-phenylindole; RT-qPCR, Real-time qPCR; IHC, immunohistochemistry; PGC-1α, peroxisome proliferators-activated receptor γ coactivator-1α; TFAM, mitochondrial transcription factor A; GSH-Px, glutathione peroxidase; TrxR, thioredoxin reductase; Dios, iodothyronine deiodinase.

## References

[1] Song C., Wang Z., Cao J., Dong Y., Chen Y. (2024). Neurotoxic mechanisms of mycotoxins: Focus on aflatoxin B1 and T-2 toxin. Environ. Pollut..

[2] Ueno Y. (1984). Toxicological features of T-2 toxin and related trichothecenes. Fundam. Appl. Toxicol..

[3] Song W., Wang Y., Huang T., Liu Y., Chen F., Chen Y., Jiang Y., Zhang C., Yang X. (2023). T-2 toxin metabolism and its hepatotoxicity: New insights on the molecular mechanism and detoxification. Environ. Pollut..

[4] Wang P., Sun L.H., Wang X., Wu Q., Liu A. (2024). Effective protective agents against the organ toxicity of T-2 toxin and corresponding detoxification mechanisms: A narrative review. Anim. Nutr..

[5] Dai C., Xiao X., Sun F., Zhang Y., Hoyer D., Shen J., Tang S., Velkov T. (2019). T-2 toxin neurotoxicity: Role of oxidative stress and mitochondrial dysfunction. Arch. Toxicol..

[6] He J., Jin H., Guo J., Li K., Jia L., Li Y., Zhang L. (2024). T-2 toxin-induced testicular impairment by triggering oxidative stress and ferroptosis. Ecotoxicol. Environ. Saf..

[7] Zhang S., Song W., Hua Z., Du J., Lucena R.B., Wang X., Zhang C., Yang X. (2024). Overview of T-2 Toxin Enterotoxicity: From Toxic Mechanisms and Detoxification to Future Perspectives. J. Agric. Food Chem..

[8] An K., Shi B., Lv X., Liu Y., Xia Z. (2024). T-2 toxin triggers lipid metabolism disorder and oxidative stress in liver of ducks. Ecotoxicol. Environ. Saf..

[9] Yang L., Yu Z., Hou J., Deng Y., Zhou Z., Zhao Z., Cui J. (2016). Toxicity and oxidative stress induced by T-2 toxin and HT-2 toxin in broilers and broiler hepatocytes. Food Chem. Toxicol..

[10] Pecoraro B.M., Leal D.F., Frias-De-Diego A., Browning M., Odle J., Crisci E. (2022). The health benefits of selenium in food animals: A review. J. Anim. Sci. Biotechnol..

[11] Surai P.F., Kochish I.I. (2019). Nutritional modulation of the antioxidant capacities in poultry: The case of selenium. Poult. Sci..

[12] Edens F.W., Sefton A.E. (2016). Organic selenium in animal nutrition—Utilisation, metabolism, storage and comparison with other selenium sources. J. Appl. Anim. Nutr..

[13] Jing J., Zeng H., Shao Q., Tang J., Wang L., Jia G., Liu G., Chen X., Tian G., Cai J. (2023). Selenomethionine alleviates environmental heat stress induced hepatic lipid accumulation and glycogen infiltration of broilers via maintaining mitochondrial and endoplasmic reticulum homeostasis. Redox Biol..

[14] Thuluvath P.J., Triger D.R. (1992). Selenium in chronic liver disease. J. Hepatol..

[15] Zhang Z., Xu J., Zhang X., Wang J., Xie H., Sun Y., Zhang Q., Chang Z., Liu Y. (2022). Protective Effect of SeMet on Liver Injury Induced by Ochratoxin A in Rabbits. Toxins.

[16] Wang T., Li H., Li Y., Li M., Zhao H., Zhang W., Zhao T., Wang Y., Wang J., Wang J. (2024). Selenomethionine supplementation mitigates fluoride-induced liver apoptosis and inflammatory reactions by blocking Parkin-mediated mitophagy in mice. Sci. Total Environ..

[17] Chen X., Zhang J., Li H., Liu W., Xi Y., Liu X. (2022). A Comprehensive Comparison of Different Selenium Supplements: Mitigation of Heat Stress and Exercise Fatigue-Induced Liver Injury. Front. Nutr..

[18] Zhang J., Zhou H., Li H., Ying Z., Liu X. (2021). Research progress on separation of selenoproteins/Se-enriched peptides and their physiological activities. Food Funct..

[19] Zhang Z., Zhang Q., Li M., Xu J., Wang J., Li M., Wei L., Lv Q., Chen X., Wang Y. (2022). SeMet attenuates AFB1-induced intestinal injury in rabbits by activating the Nrf2 pathway. Ecotoxicol. Environ. Saf..

[20] Gao S., Wang K., Xiong K., Xiao S., Wu C., Zhou M., Li L., Yuan G., Jiang L., Xiong Q. (2023). Unraveling the Nrf2-ARE Signaling Pathway in the DF-1 Chicken Fibroblast Cell Line: Insights into T-2 Toxin-Induced Oxidative Stress Regulation. Toxins.

[21] Wu Q.H., Dohnal V., Huang L.L., Kuca K., Yuan Z.H. (2010). Metabolic pathways of trichothecenes. Drug Metab. Rev..

[22] Königs M., Mulac D., Schwerdt G., Gekle M., Humpf H.U. (2009). Metabolism and cytotoxic effects of T-2 toxin and its metabolites on human cells in primary culture. Toxicology.

[23] Li J., Bai Y., Ma K., Ren Z., Li J., Zhang J., Shan A. (2022). Dihydroartemisinin alleviates deoxynivalenol induced liver apoptosis and inflammation in piglets. Ecotoxicol. Environ. Saf..

[24] Yam C., Zhao M., Hayashi K., Ma H., Kishimoto H., McElroy M., Bouvet M., Hoffman R.M. (2010). Monotherapy with a Tumor-Targeting Mutant of *S. typhimurium* Inhibits Liver Metastasis in a Mouse Model of Pancreatic Cancer. J. Surg. Res..

[25] Wang L., Cheng D., Wang H., Di L., Zhou X., Xu T., Yang X., Liu Y. (2009). The hepatoprotective and antifibrotic effects of *Saururus chinensis* against carbon tetrachloride induced hepatic fibrosis in rats. J. Ethnopharmacol..

[26] Ramaiah S.K. (2007). A toxicologist guide to the diagnostic interpretation of hepatic biochemical parameters. Food Chem. Toxicol. Int. J. Publ. Br. Ind. Biol. Res. Assoc..

[27] Green R.M., Flamm S. (2002). AGA technical review on the evaluation of liver chemistry tests. Gastroenterology.

[28] Sies H. (1997). Oxidative stress: Oxidants and antioxidants. Exp. Physiol..

[29] Frijhoff J., Winyard P.G., Zarkovic N., Davies S.S., Stocker R., Cheng D., Knight A.R., Taylor E.L., Oettrich J., Ruskovska T. (2015). Clinical Relevance of Biomarkers of Oxidative Stress. Antioxid. Redox Signal..

[30] Fang H., Wu Y., Guo J., Rong J., Ma L., Zhao Z., Zuo D., Peng S. (2012). T-2 toxin induces apoptosis in differentiated murine embryonic stem cells through reactive oxygen species-mediated mitochondrial pathway. Apoptosis.

[31] Chen F., Wang Y., Chen Y., Fan J., Zhang C., He X., Yang X. (2023). JNK molecule is a toxic target for IPEC-J2 cell barrier damage induced by T-2 toxin. Ecotoxicol. Environ. Saf..

[32] Lauridsen C. (2019). From oxidative stress to inflammation: Redox balance and immune system. Poult. Sci..

[33] Dikalov S.I., Dikalova A.E. (2019). Crosstalk Between Mitochondrial Hyperacetylation and Oxidative Stress in Vascular Dysfunction and Hypertension. Antioxid. Redox Signal..

[34] Peoples J.N., Saraf A., Ghazal N., Pham T.T., Kwong J.Q. (2019). Mitochondrial dysfunction and oxidative stress in heart disease. Exp. Mol. Med..

[35] Chan S.H.H., Chan J.Y.H. (2017). Mitochondria and Reactive Oxygen Species Contribute to Neurogenic Hypertension. Physiology.

[36] Scarpulla R.C. (2011). Metabolic control of mitochondrial biogenesis through the PGC-1 family regulatory network. Biochim. Biophys. Acta.

[37] Scarpulla R.C., Vega R.B., Kelly D.P. (2012). Transcriptional integration of mitochondrial biogenesis. Trends Endocrinol. Metab..

[38] Ma S., Zhao Y., Sun J., Mu P., Deng Y. (2017). miR449a/SIRT1/PGC-1α Is Necessary for Mitochondrial Biogenesis Induced by T-2 Toxin. Front. Pharmacol..

[39] Wredenberg A., Wibom R., Wilhelmsson H., Graff C., Wiener H.H., Burden S.J., Oldfors A., Westerblad H., Larsson N.G. (2002). Increased mitochondrial mass in mitochondrial myopathy mice. Proc. Natl. Acad. Sci. USA.

[40] Vanasco V., Saez T., Magnani N.D., Pereyra L., Marchini T., Corach A., Vaccaro M.I., Corach D., Evelson P., Alvarez S. (2014). Cardiac mitochondrial biogenesis in endotoxemia is not accompanied by mitochondrial function recovery. Free Radic. Biol. Med..

[41] Sun X.H., Lv M.W., Zhao Y.X., Zhang H., Ullah Saleem M.A., Zhao Y., Li J.L. (2023). Nano-Selenium Antagonized Cadmium-Induced Liver Fibrosis in Chicken. J. Agric. Food Chem..

[42] Jiang Y., Qian Y., Hong H., Gao X., Liu W., Jin Q., Chen M., Jin Z., Liu Q., Wei Z. (2023). Morin protects chicks with T-2 toxin poisoning by decreasing heterophil extracellular traps, oxidative stress and inflammatory response. Br. Poult. Sci..

[43] Dong B., Jiang Y., Shi B., Zhang Z., Zhang Z. (2024). Selenomethionine alleviates decabromodiphenyl ether-induced oxidative stress and ferroptosis via the NRF2/GPX4 pathway in the chicken brain. J. Hazard. Mater..

[44] Bellinger F.P., Raman A.V., Reeves M.A., Berry M.J. (2009). Regulation and function of selenoproteins in human disease. Biochem. J..

