# Supplementary material for: Selenomethionine Counteracts T-2 Toxin-Induced Liver Injury by Mitigating Oxidative Stress Damage Through the Enhancement of Antioxidant Enzymes
Source: Antioxidants (Basel). 2025 Jul 15;14(7):866. doi: 10.3390/antiox14070866 (PMC12292169; doi:10.3390/antiox14070866)
Supplement: Supplementary file 1 [file antioxidants-14-00866-s001.zip › antioxidants-3668493-supplementary.pdf]

Supplementary materials

Table S1. Primer sequences used for real-time quantitative PCR

Primer	Sequence (5'-3')	NCBI accession
GAPDH-F	GATGGGCGTGAACCATGAGA	OX439033.1
GAPDH-R	GATGCCGAAGTGGTCATGGA	
GPX1-F	TCAGTTCGGACACCAGAATG	NM_001329527.1
GPX1-R	TCTCACCATTCACTTCGCACT	
CAT-F	ATGGTCACCGGCACATGAAT	LC706448.1
CAT-R	GCCCTGGTCGGTCTTGTAAT	
SOD-F	TGCAGGGAACCATCCACTTCG	OX439031.1
SOD-R	CCCATGCTGGCCTTCAGTTAATC	
IL-1β-F	AAATACCTGTGGCCTTGGGC	XM_006498795.5
IL-1β-R	CTTGGGATCCACACTCTCCAG	
IL-6 -F	TGATGGATGCTACCAAACTGGA	NM_001314054.1
IL-6 -R	TGTGACTCCAGCTTATCTCTTGG	
IL-10-F	ACAACATACTGCTAACCGACTCC	LR761010.1
IL-10-R	TTCATTCATGGCCTTGTAGACAC	
TNF-α-F	AGCCGATGGGTTGTACCTTG	NM_001278601.1
TNF-α-R	AGTACTTGGGCAGATTGACCTC	
TGF-β1-F	CCCTGTATTCCGTCTCCTT	XM_036152883.1
TGF-β1-R	ATTCCTGGCGTTACCTTG	
